# Preconception, Interconception, and reproductive health screening tools: A systematic review

**DOI:** 10.1111/1475-6773.14123

**Published:** 2023-01-06

**Authors:** Megan Ren, Hannah Shireman, Emily White VanGompel, Jennifer K. Bello, Francesca Carlock, Ashley McHugh, Debra Stulberg

**Affiliations:** ^1^ Pritzker School of Medicine University of Chicago Chicago Illinois USA; ^2^ Department of Family Medicine University of Chicago Chicago Illinois USA; ^3^ Northshore University Healthsystem Evanston Illinois USA; ^4^ Department of Family and Community Medicine Saint Louis University St Louis Missouri USA

**Keywords:** interconception care, preconception care, pregnancy intention screening, primary care, reproductive counseling, reproductive life plan, systematic review

## Abstract

**Objective:**

To identify and describe the standardized interconception and preconception screening tools for reproductive health needs that are applicable in general outpatient clinical practice.

**Data Sources and Study Setting:**

This systematic review identifies research on pregnancy intention screening and counseling tools, and standardized approaches to preconception and interconception care. We focus on tools designed for clinical settings, but also include research tools with potential for clinical implementation. These tools may include a component of contraceptive counseling, but those focusing solely on contraceptive counseling were excluded. Data were collected from studies done in the United States between January 2000 and March 2022.

**Study Design:**

We performed a systematic literature search to generate a list of unique tools, assessed the quality of evidence supporting each tool, and described the peer‐reviewed clinical applications of each. We used the Mixed Methods Appraisal Tool to appraise the quality of individual studies.

**Data Collection/Extraction Methods:**

We searched PubMed, Web of Science, and CINAHL databases for standardized preconception and interconception health screening tools published in English from January 2000 through March 2022. We used keywords “preconception care,” “interconception care,” “family planning,” “contraception,” “reproductive health services,” and “counseling.” Utilizing the Preferred Reporting Items for Systematic Reviews guidelines, we screened titles and abstracts to identify studies for full text review.

**Principal Findings:**

The search resulted in 15,399 studies. After removing 4172 duplicates, we screened 11,227 titles/abstracts and advanced 207 for full‐text review. From these, we identified 53 eligible studies representing 22 tools/standardized approaches, of which 10 had evidence from randomized clinical trials. These ranged widely in design, setting, and population of study.

**Conclusions:**

Clinicians have a choice of tools when implementing standard reproductive screening services. A growing body of research can inform the selection of an appropriate tool, and more study is needed to establish effects on long‐term patient outcomes.

**What is known on this topic**

- A barrier to preconception and interconception health screening in general outpatient practices is the number of competing clinical needs
- Many standardized tools exist that proactively screen for these reproductive health needs
- Clinicians seeking guidance on these tools lack a structured review of different approaches

**What this study adds**

- This study provides a structured review of different approaches to preconception and interconception health screening
- The search yielded 53 eligible studies representing 22 tools/standardized approaches, of which 10 had evidence from randomized clinical trials. Tools ranged widely in design, setting, and population of study.

## 1 INTRODUCTION

Many factors associated with perinatal morbidity and mortality are chronic conditions that begin prior to the start of pregnancy. To address these upstream risks and help people control the timing and conditions of pregnancy, the Centers for Disease Control and Prevention (CDC) and the American College of Obstetricians and Gynecologists (ACOG) recommend routine preconception health counseling, defined as “the health of people during their reproductive years, or the years they can have a child”.^1^, ^2^ However, only 14% of ambulatory visits in the United States include any preconception or contraceptive counseling. Among women at particular risk of increased pregnancy‐related morbidity, including those with a recent birth who had diabetes, hypertension, or both prior to becoming pregnant, fewer than half report receiving preconception health counseling.^3^, ^4^ Additionally, low‐income women are more likely to receive reproductive health services in primary care (vs. dedicated women's health settings) and are disproportionately cared for in federally qualified health centers (FQHCs).^5^, ^6^ Women who have Medicaid insurance or are uninsured have been shown to have increased rates of adverse birth outcomes,^7^ hence, it is particularly important that general outpatient practices have a means of identifying those that need preconception/interconception care. In this paper, we use “general outpatient practice” to include primary care and other outpatient practices not specifically designed for family planning.

Patients have expressed interest in receiving preconception/interconception health screening and counseling from their primary care provider (PCP).^8^, ^9^ However, numerous competing demands, lack of preconception health knowledge by both patients and clinicians, and lack of ownership in delivery of preconception care are well‐documented barriers.^10^, ^11^ Often, clinicians do not even recognize that a patient would be eligible for preconception care.^12^ Despite the routine use of screening tools to identify other health needs such as depression, screening for preconception and interconception health needs is not widely adopted and there is a lack of consensus on the best approach.^13^

To help clinicians in overcoming barriers to incorporating preconception counseling, we conducted this systematic review of preconception, interconception, and pregnancy intention screening tools and standardized (i.e., replicable) approaches relevant to general outpatient practice in the United States. Many of these standardized approaches involved the use of a specific tool, such as a screening question or form, but any study adopting a standardized approach (with or without a specific tool) was included in this review. In describing specific studies, we mirrored the language authors chose for framing their research (i.e., “interconception counseling” vs. “reproductive counseling”, “pregnant person” vs. “woman”); however, we recognize that people of all genders, including nonbinary and transgender individuals, can become pregnant and give birth. The study's objective is to describe these standardized approaches and tools, the settings in which they have been studied, and the published findings about their uses and limitations.

## 2 METHODS

### 2.1 Search strategy and screening

**FIGURE 1:**
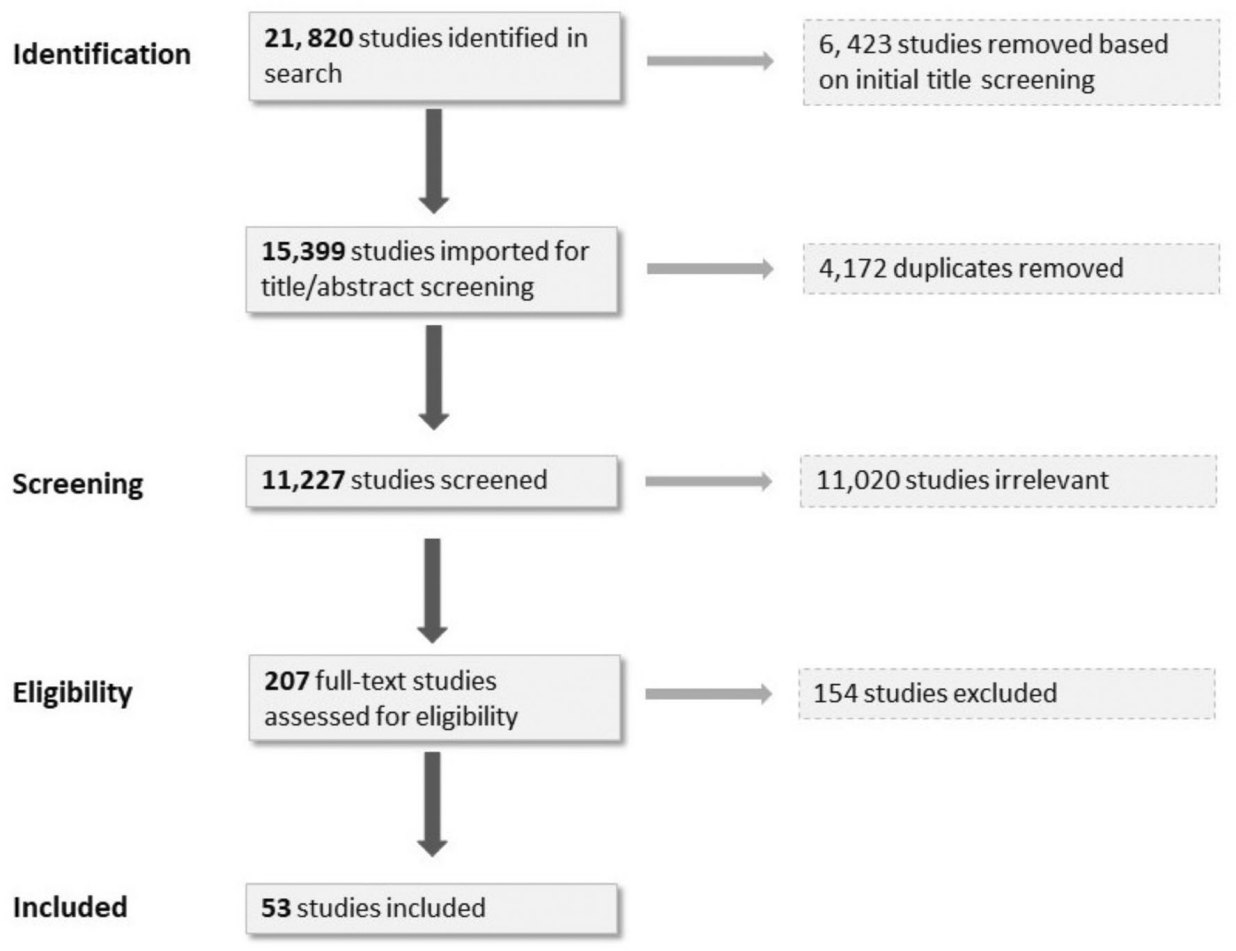
PRISMA diagram of included studies. Flow diagram of our literature search from three databases (PubMed, Web of Science, CINAHL).

We conducted a structured search of the PubMed, Web of Science, and CINAHL databases for standardized preconception and interconception health screening tools published in English between January 1, 2000 and March 1, 2022. Our search strategy utilized a combination of phrases and keywords, including: “preconception care,” “interconception care,” “family planning,” “contraception,” “reproductive health services,” and “counseling.” The University of Chicago biomedical librarians provided guidance throughout the development of our search strategy and a complete list of search terms and criteria can be found in (Table S1). To maintain a high degree of search sensitivity, no additional database filters were applied. Studies with titles clearly indicating that they were conducted outside the United States were removed from the results to be screened. The search process was conducted from March to April 2022 and resulted in a total of 15,399 studies. After removing 4172 duplicates, we reviewed the remaining 11,227 (Figure 1).

Our inclusion criteria centered on standardized approaches or tools with potential for implementation within clinical settings in the United States. Tools tested in family planning clinics were accepted if they were also applicable in general outpatient settings. To ensure included studies outline tools with potential for clinical implementation, we excluded approaches that required resources beyond the typical clinical setting and those that focused solely on skills development for clinical staff or students. We considered contraceptive needs assessment and counseling a component of preconception and interconception care. Given our study's objective, we excluded studies that focused exclusively on contraceptive use, continuation, or method choice, unless the tool had clear applicability for preconception care more broadly defined. Table 1 provides the full list of inclusion and exclusion criteria used.

**TABLE 1:** Inclusion and exclusion criteria

Inclusion criteria	Exclusion criteria
Study Publication between January 2000 and March 2022 Original research articles Randomized control trials, observational assessments	Study Studies that take place outside the United States Non‐English language articles Systematic, scoping, or literature reviews; dissertations, editorials, commentaries, conference abstracts
Tool/Approach Addresses preconception/ interconception care Designed for clinical setting Research tools with potential for clinical implementation	Tool/Approach Exclusively about patient choice of contraception method or use of contraception Curricula for clinicians or clinical staff Requires resources (including personnel) beyond a typical clinical setting

Using the Covidence platform to manage and track our screening process, each title/abstract was reviewed by two study team members to determine whether the study could be eligible for inclusion.^14^, ^15^ If the first two reviewers' eligibility decisions conflicted, a separate reviewer performed a third screening to determine inclusion eligibility. Once initial eligibility was assessed, full text manuscripts were reviewed to determine final inclusion. Full text review was similarly conducted by two separate authors. If eligibility assessments conflicted, the two reviewing authors discussed the article and made a final decision together.

### 2.2 Data abstraction

We exported the included studies from Covidence to Excel and identified the following characteristics of each: the study objectives, the setting and/or population studied, the primary methods, and the key findings.

### 2.3 Quality assessment

To assess the methodological quality of included studies, we used the Mixed Methods Appraisal Tool (MMAT) designed for critical appraisal of qualitative research, randomized controlled trials, non‐randomized studies, quantitative descriptive studies, and mixed methods studies.^16^ Each study was independently assessed by two reviewers. Reviewers did not assess studies for which they were included as authors. For each MMAT question, reviewers responded “Yes” if the study met relevant criteria, “No” if the study did not meet relevant criteria, or “Cannot tell” if the information reported was not adequate to determine whether criteria were met. Of the 53 included studies, 5 were qualitative, 15 were randomized controlled trials, 16 were non‐randomized, 11 were quantitative descriptive, 5 were mixed‐methods studies, and 1 was a corrigendum.

To identify the most rigorous level of research supporting individual tools/approaches, we assessed the overall quality and robustness of the body of evidence available for each. Based on the list of identified studies about each tool, we categorized each as: (1) tested with a randomized controlled trial (RCT); (2) observational study/studies, with assessment of patient outcomes (which could include rates of counseling or patient‐reported satisfaction with care); these encompassed cohort studies and case–control studies; (3) clinical feasibility/acceptability/patient experience studies or validated measure. We assigned a tool the highest level of published quality, for example, we assigned a study that had both a randomized control trial and observational studies as “tested with RCT” based on the levels of evidence as defined by the Oxford Centre of Evidence‐Based Medicine.^17^

## 3 RESULTS

### 3.1 Search outcome

Of the 11,227 studies that underwent title and abstract screening, we excluded 11,020 and advanced 207 for full‐text review. We excluded an additional 154 studies during full‐text review, yielding 53 for final inclusion and analysis (Figure 1).

### 3.2 Article characteristics

Table 2 provides a descriptive summary of the studies included in this review. The majority of articles are published after 2010. Methods included feasibility assessments, pre/post comparisons, and randomized controlled trials (RCTS). The 53 studies encompassed 22 distinct tools or approaches (Table 3), with a focus on different populations and implementation settings.

**TABLE 2:** Description of included studies

Citation number	Title	Author and year	Location/Practice setting	Study purpose/Objective	Study methods	Study results
9	Women's Perspectives on Reproductive Health Services in Primary Care	Manze et al. (2020)	Primary care settings in New York state (urban, suburban, and rural)	Understand patient perspectives on receipt of reproductive health (RH) services in primary care settings	Explored participants' preferences for RH services from primary care providers and asked their opinions on three pregnancy intention screening and reproductive health needs assessment questions. Transcripts were thematically analyzed.	Participants were receptive to the availability of RH services in primary care and had the most positive response to the proposed question “Can I help you with any reproductive health services today, such as birth control or planning for a healthy pregnancy?”
12	Perceptions of a Reproductive Health Self‐Assessment Tool (RH‐SAT) in an Urban Community Health Center	Bello et al. (2013)	Urban community health center	Examine the impact of a novel reproductive health self‐assessment tool (RH‐SAT) on reproductive health counseling	Providers were trained on preconception and contraceptive guidelines. Semi‐structured interviews were conducted to assess perceptions of the tool with 22 patients and with all 15 providers at the clinic. Transcripts were thematically analyzed using a grounded theoretical approach.	This RH‐SAT was acceptable and useful to patients, prompting them to address topics they otherwise did not think to discuss.
21	Motivational Intervention to Reduce Rapid Subsequent Births to Adolescent Mothers: A Community‐Based Randomized Trial	Barnet et al. (2009)	Urban prenatal clinics serving low‐income, predominantly African‐American communities	Determine the effectiveness of a computer‐assisted motivational intervention (CAMI) in preventing rapid subsequent birth to adolescent mothers	Participants were randomly assigned to three groups: *CAMI*+ (*n* = 80) received a multi‐component home‐based intervention; *CAMI‐only* (*n* = 87) received a single component home‐based intervention; *control* (*n* = 68) received usual care. Repeat birth by 24 months' postpartum was measured with birth certificates.	Completing two or more CAMI sessions significantly reduced the risk of repeat birth in both groups: CAMI+ (HR = 0.40; 95% CI, 0.16–0.98) and CAMI‐only (HR = 0.19; 95% CI, 0.05–0.69).
22	A Cluster Randomized Controlled Trial of the MyFamilyPlan Online Preconception Health Education Tool	Batra et al. (2018)	Urban academic medical center in California	Evaluate whether exposure to MyFamilyPlan‐a web‐based preconception health education module‐changes the proportion of women discussing reproductive health with providers at well‐woman visits	Intervention participants completed online module before a well‐woman visit; control participants reviewed standard preconception health materials. The primary outcome was discussion of reproductive health with the physician. Secondary outcomes were folic acid use, contraceptive method initiation/change, and self‐efficacy score.	After adjusting for covariates and cluster, exposure to MyFamilyPlan was the only variable significantly associated with an increase in the proportion of women discussing reproductive health with providers (odds ratio: 1.97, 95% confidence interval: 1.22–3.19).
23	Perceptions of a Spanish language Reproductive Health Self‐assessment Tool Among Spanish‐Speaking Women at a Federally Qualified Health Center	Bello et al. (2020)	Federally qualified health center in Chicago	Overcome barriers to contraceptive and preconception care among Latina women using a reproductive health self‐assessment tool (RH‐SAT)	Twenty Spanish‐speaking women at a federally qualified health center in Chicago received the RH‐SAT before their visit then completed a phone interview about their perceptions of the RH‐SAT. Transcripts were thematically analyzed using a modified grounded theoretical approach.	Participants believed that the RH‐SAT was easy to use and its content was useful and felt that it provided new information about preparing for pregnancy and contraception. They felt that the RH‐SAT prompted them to self‐reflect and ask questions not previously considered and that it could help overcome barriers to discussing reproductive health.
24	Improving Preconception Care	Bernstein et al. (2000)	Outpatient gynecology clinic at an inner city hospital in New York	Assess the ability of a preconception care provider education program to change knowledge/attitudes and improve the delivery of preconception care	Pre‐intervention chart review of a convenience sample of women (*n* = 100) and provider survey. The two‐part intervention was then carried out, followed by a post‐intervention chart review of a second convenience sample (*n* = 100) and repeat provider survey to assess change in knowledge and attitudes toward preconception care.	Documentation of screening in almost all categories was significantly improved. The greatest improvements were noted in complete screening for medical risk factors (15%–44%), over‐the‐counter and prescription medication use (10%–70% and 30%–77%, respectively), domestic violence (from 10% to 57%), and nutrition (from 9% to 50%).
25	Promotion of Preconception Care Among Adolescents and Young Adults by Conversational Agent	Bickmore et al. (2020)	National sample of 528 AA/Black women aged 18–34 years	Assess the acceptance, usability, and use of conversational agent “Gabby” to screen women and address preconception risks, and to compare use across age groups	Secondary data analysis on the Gabby system use and self‐reported usability and satisfaction of the 79 women aged 18–25 years randomized to the intervention group, compared with the 183 women aged 26–34 years in the intervention group.	Most participants found Gabby easy to use and said that they would recommend the system. There were no significant differences in use or acceptability between the younger versus older age participants. Both groups used Gabby a median of six times over a year.
26	Development and Pilot Testing of a Patient‐Centered Web‐Based Reproductive Decision Support Tool for Primary Care	Callegari et al. (2021)	Primary care centers in the veterans administration	Assess feasibility and acceptability of MyPATH tool to help women veterans achieve reproductive health goals	Providers and staff received a brief introduction to MyPath. Patients scheduled to see providers in the intervention phase used MyPath on an iPad in the waiting room. Researchers measured acceptability, feasibility, discussions about pregnancy and/or contraceptive needs, and contraceptive decision quality by a survey of participants and providers.	Nearly all participants who used MyPath reported that they learned new information (97%) and would recommend it to others (93%). No providers reported that MyPath significantly increased workload. More intervention participants reported having discussions about reproductive needs in their visit compared to controls (93% vs. 68%; *p* = 0.02). Intervention participants also experienced greater increases in knowledge and self‐efficacy.
27	A Novel Approach to Postpartum Contraception: A Pilot Project of Pediatricians' Role During the Well‐Baby Visit	Caskey et al. (2016)	Well‐baby visits at an academic center	Test the feasibility and acceptability of having pediatric residents administer a simplified Reproductive Life Plan Tool (RLPT) with postpartum women during routine infant care	Pediatric resident physicians used the RLPT with mothers of infants ≤16‐weeks during WBVs. The RLPT prompts physicians to ask about contraceptive needs and offer referrals. Residents participated in a feedback session and survey to assess acceptance and perceived feasibility of using the RLPT during routine care.	Use of the RLPT is generally feasible during routine infant care and acceptable to pediatric resident physicians with recognition of challenges to implementation. Acceptance of a referral was low among postpartum women in this pilot study.
28	Randomized Efficacy Trial of Early Preconception Counseling for Diabetic Teens (READY‐Girls)	Charron‐Prochownik et al. (2008)	Diabetes clinics	Develop and assess the feasibility of an early preconception counseling program for adolescents called READY‐Girls (Reproductive‐health Education and Awareness of Diabetes in Youth for Girls)	Adolescent girls (*n* = 53) with type 1 diabetes were randomized into groups receiving a video, a book, or standard care (control) and given one comprehensive session. Outcomes were assessed at baseline, immediately after, and at 3 months.	Teens who received the CD and those who received the book demonstrated significant improvement (over 3 months) in knowledge, perceived benefits of both receiving preconception counseling and using effective family planning, and perceived more support with reproductive health issues.
29	Mother‐Daughter Dyadic Approach for Starting Preconception Counseling at Puberty in Girls with Diabetes	Charron‐Prochownik et al. (2014)	Diabetes clinics at two university hospitals	Feasibility study to explore mothers' and daughters' awareness and knowledge of diabetes and pregnancy, and preconception counseling; compare mother‐daughter responses using dyadic analyses	Mixed‐method study with 10 mothers of daughters with type 1 diabetes. Mothers were interviewed using three open‐ended questions and completed knowledge and support questionnaires. Their responses were compared to those of their daughters who were participating in a randomized controlled trial with READY‐Girls.	Mothers and daughters both reported little knowledge of preconception health. Mothers perceived giving more support than their daughters described receiving.
30	Long‐Term Effects of the Booster‐Enhanced READY‐Girls Preconception Counseling Program on Intentions and Behaviors for Family Planning in Teens with Diabetes	Charron‐Prochownik et al. (2013)	Diabetes clinics at two university hospitals	Examine 12‐month effects of a booster‐enhanced preconception counseling program (READY‐Girls) on family planning for teen girls with type 1 and type 2 diabetes	Participants 13–19 years of age (*n* = 109) were randomized to a standard care control group or intervention group that received counseling over three consecutive clinical visits. Pre/post data were collected at baseline, 3‐ and 6‐month booster sessions, and a 12‐month follow‐up visit.	Intervention group participants retained greater preconception knowledge and held stronger intentions to seek care prior to a future pregnancy, compared to control group participants.
31	A Theory‐Based Reproductive Health and Diabetes Instrument	Charron‐Prochownik et al. (2006)	Four university‐based medical centers with pediatric diabetes clinics	Determine psychometric properties of scales within the theoretically based Reproductive Health Attitudes and Behavior (RHAB) instrument for examining preconception planning of young women with diabetes	Psychometrics were examined on data from a telephone interview on this 48‐item instrument using a sample of 87 female adolescents with diabetes from four medical centers.	Overall, the major factors (scales) clustered according to theoretical underpinning. Most Cronbach alphas were moderate (0.60–0.83). RHAB appears to have acceptable levels of validity/reliability for use with female adolescents with diabetes.
32	Effectiveness of a Pediatric Primary Care Intervention to Increase Maternal Folate Use: Results from a Cluster Randomized Controlled Trial	Chilukuri et al. (2018)	Four urban pediatric practices in Baltimore, Maryland	Assess the impact of a preconception health intervention (15‐item screening and counseling and 90‐day multivitamin supply) on folate use among mothers bringing infants to pediatric primary care	Clinicians (*n* = 45) were randomized into an intervention or control group. In the control, patients received community resource handouts, and both groups received 90‐day multivitamin (MVI) supply. Mothers completed interviews at baseline and 6‐month follow‐up to assess daily use of folate.	Among all participants, daily vitamin intake increased from baseline to 6‐month follow‐up (33.8% vs. 42.6%; *p* = 0.016). After adjustment for covariates and clustered design, there was an augmented effect in the intervention versus control group (aOR, 2.04; 95% CI, 1.04–3.98).
33	Interventions to Increase Multivitamin Use Among Women in the Interconception Period: An IMPLICIT Network Study	DeMarco et al. (2021)	Family medicine clinical sites	Measure effects of interventions to improve folate supplementation in women prior to conception	Mothers at WCVs were asked about multivitamin (MVI) use and family planning methods and were offered appropriate interventions. Logistic regression was used to estimate the effect of interventions on subsequent MVI use.	37.7% of mothers reported not using MVIs and 64% received an intervention to improve MVI use. Mothers were more likely to report taking an MVI at the subsequent WCV if they received advice to take MVIs (OR 1.64) or directly received MVI samples (OR 3.09).
34	Utility of Reproductive Life Plans in Identification of Potentially Teratogenic Medication Use: A Pilot Study	DiPietro et al. (2018)	Toledo‐Lucas County healthy start program	Determine the utility of reproductive life plans (RLPs) as tools to identify women using potentially teratogenic medications	Retrospective review of RLPs completed by women receiving services. The medication section of each RLP was reviewed to determine: if it was completed; categories of medications reported (prescription, over‐the‐counter, vitamin/herbal); potential teratogens; contraception method.	Most RLPs (75%) had the medication section completed. 8% showed the patient taking a potential teratogen, and < 30% of these showed contraceptive use.
35	Integrating Reproductive Planning with Primary Health Care: An Exploration Among Low‐Income, Minority Women and Men	Dunlop et al. (2010)	Publicly‐funded primary care clinics in metropolitan Atlanta	Explore the acceptability and utility of integrating an assessment of reproductive plans into primary care encounters	144 purposively sampled African‐American or Hispanic participants were assessed for their desire to have a child and their contraceptive practices via a reproductive plans questionnaire. Patients' responses were attached to the medical record for provider use. After the encounter, semi‐structured interviews elicited patients' opinions about the questionnaire.	Most women (81%) and many men (42%) reported that the reproductive plans assessment was important to their encounter, with variation in the reason according to reported desire for a child. >45% who reported never wanting a child or not wanting a child for at least one year were at‐risk for unintended pregnancy. Many patients reported uncertainty about desiring a child.
36	Acceptability and Potential Impact of Brief Preconception Health Risk Assessment and Counseling in the WIC Setting	Dunlop et al. (2013)	A Women, Infants, and Children (WIC) program in Clayton County, Georgia	Determine the reproductive risks of women using the Special Supplemental Nutrition Program for Women, Infants, and Children and the acceptability of delivering preconception screening and counseling with the WIC encounter	Participants were administered a risk assessment questionnaire to determine topics for brief counseling. Following counseling, participants completed a semi‐structured interview. The risk assessment questionnaire was analyzed quantitatively; transcripts from the interviews were analyzed thematically.	Reproductive risks were prevalent: history of unintended pregnancy (27%), sexually transmitted infection (49%), inadequate folic acid use (66%), intimate partner violence (47%), tobacco use (21%), binge drinking (10%), and illicit drug use (5%). Most WIC clients found the preconception risk assessment and counseling acceptable and important.
37	Change in Women's Knowledge of General and Personal Preconception Health Risks Following Targeted Brief Counseling in Publicly Funded Primary Care Settings	Dunlop et al. (2013)	Five publically funded clinics in greater metropolitan Atlanta area	Explore knowledge of general and personal preconception health risks among women in publicly funded clinics and whether brief counseling can improve knowledge	Patients in the intervention group received targeted brief counseling based upon risks identified via preconception health risk assessment. McNemar's test was used to compare proportion of women in each cohort who correctly answer questions of preconception health knowledge pre‐encounter versus 3–6 months post‐encounter.	Women in the intervention cohort experienced a significant increase in knowledge related to preconception health from baseline to 3–6 months post‐encounter. Among women with chronic medical conditions, those in the intervention cohort significantly increased their knowledge that the condition could lead to problems in pregnancy (+43%) relative to the lesser improvement in knowledge observed for those in the comparison cohort (+4%) (*p* < 0.05).
38	Facilitators of and Barriers to Successful Implementation of the One Key Question() Pregnancy Intention Screening Tool	Ferketa et al. (2022)	One OB/GYN and one family medicine clinic in suburbs near Chicago	Explore the barriers and facilitators of OKQ implementation to better understand how to best implement the tool across different settings	Staff and clinicians completed surveys and qualitative interviews about their experiences with the tool. Thematic analysis of the interviews was informed by the Consolidated Framework for Implementation Research (CFIR).	Facilitators of implementation included simplicity of OKQ, engagement of clinical leadership, and compatibility between OKQ's goals and those of staff and clinicians. Time constraints were a concern but OKQ had minimal impact on clinical workflow during implementation. In Family Medicine, barriers also included OKQ distracting from the visit agenda, and concerns about screening patients at every visit.
39	Impact of a Preconception Counseling Program for Teens with Type 1 Diabetes (READY‐Girls) on Patient‐Provider Interaction, Resource Utilization, and Cost	Fischl et al. (2010)	Diabetes clinics at two university hospitals	Evaluate the impact of a preconception counseling program tailored for teens with type 1 diabetes on cognitive, psychosocial, and behavioral outcomes and to assess its cost‐effectiveness	Teens with type 1 diabetes were randomized into the READY‐Girls intervention (*n* = 43) or standard care (*n* = 45) groups. During three clinical visits, intervention subjects viewed a video, read a book, and met with a nurse. Program effectiveness was measured by knowledge, attitudes, intentions, and behaviors reported by patients at baseline, before and after viewing materials, and at 9 months.	READY‐Girls participants had significantly greater increases in preconception knowledge and perceived benefits of preconception care, compared to controls; effects were sustained at 9 months.
40	Interconception Care for Mothers at Well Child Visits After Implementation of the IMPLICIT Model	Frayne et al. (2021)	Family medicine clinical sites	Evaluate whether IMPLICIT led to practice change, from the patient perspective; give insight into maternal screening at well‐child visits (WCVs); uncover new questions on how to implement this care sustainably and effectively	Mothers accompanying their child at 12‐ and 24‐month WCVs were surveyed pre‐ and post‐implementation to assess health history, behaviors, and whether their child's physician addressed maternal depression, tobacco use, family planning, and folic acid. Logistic regression was used to estimate the effect of the intervention, adjusting for demographics and insurance.	Mothers were more likely to report discussions with their child's doctor post‐intervention for family planning (31% pre to 86% post; aOR 18.65), depression screening (63%–85%; aOR 5.22), and folic acid supplementation (53%–68%; aOR 2.54). Among mothers who smoked, the percentage that reported their child's doctor recommended cessation increased from 56% to 75% (aOR = 3.66).
41	Using Health Information Technology to Engage African American Women on Nutrition and Supplement Use During the Preconception Period	Gardiner et al. (2020)	Convenience sample of 100 women (18–34 years) who self‐identified as AA/Black, from 20 states and the District of Columbia	Study the impact of conversational agent, “Gabby” in 13 domains of preconception care among 528 non‐pregnant AA/Black women. This analysis is restricted to 480 women who reported at least one of the 10 risks related to nutrition and dietary supplement use	Self‐reported stage of change data at baseline, 6, and 12 months was collected and used to construct two primary outcomes: the self reported percentage of preconception care risks from the nutrition domain that (1) progressed forward on the stage of change scale, and (2) reached the action or maintenance stage of change.	After 6 months, women using Gabby (a) reported progressing forward on the stage of change scale for, on average, 52.9% (SD, 35.1%) of nutrition and supplement risks compared to 42.9% (SD, 35.4) in the control group (IRR 1.22, 95% CI 1.03–1.45, *p* = 0.019); and (b) reported achieving the action and maintenance stage of change for, on average, 52.8% (SD 37.1) of the nutrition and supplement risks compared to 42.8% (SD, 37.9) in the control group (IRR 1.26, 96% CI 1.08–1.48, *p* = 0.004).
42	Pregnancy Risk Screening and Counseling for Women Veterans: Piloting the One Key Question in the Veterans Healthcare Administration	Gawron et al. (2021)	Primary care clinic in Salt Lake City, Utah	Assess veterans Health careaAdministration provider preferences on One Key Question (OKQ) implementation, identification of veterans' reproductive needs, and the effect of training on documentation in a women's primary care clinic	Researchers hosted OKQ training sessions for providers and staff, audio recorded group discussions on implementation barriers, and explored themes. Women veterans presenting for a primary care provider (PCP) visit self‐completed a paper screening tool. A pre‐post analysis was conducted to measure for changes in family planning documentation.	Veterans identified reproductive needs via the OKQ screening tool, but provider documentation did not reflect changes in care following training.
43	Beyond intent: Exploring the association of contraceptive choice with questions about Pregnancy Attitudes, Timing and How important is pregnancy prevention (PATH) questions	Geist et al. (2019)	Family planning health centers in Salt Lake County	Explore women's responses to PATH questions (Pregnancy Attitudes, Timing and How important is pregnancy prevention) about hypothetical pregnancies and associations with contraceptive method among new contraceptive clients	New contraceptive clients were provided no‐cost contraception for 1 year at family planning health centers. Researchers used Poisson regression to explore the association between survey‐adapted PATH questions and contraceptive method selection among 3121 clients.	Pregnancy timing and happiness about hypothetical pregnancies was associated with contraceptive method selection. Clients who wanted to wait >5 years, those who never wanted to become pregnant, and those who were uncertain were all more likely to select IUDs and implants than women who wanted to become pregnant within 5 years. Greater happiness was associated with lower chance of choosing an IUD or implant (PR 0.98; 95% CI 0.96–0.999).
44	Desire to Avoid Pregnancy and Contraceptive Use Among Female Methadone Patients in Los Angeles	Gipson et al. (2021)	Methadone clinics in Los Angeles	Collect information on pregnancy preferences and contraceptive use among women attending methadone treatment	Researchers used the Desire to Avoid Pregnancy (DAP) scale to capture women's pregnancy preferences (*n* = 46). They conducted factor analysis, descriptive analyses, and linear and logistic regressions to assess the DAP scale and to examine relationships between pregnancy preferences, sociodemographic characteristics, and contraceptive use.	Women expressed a range of pregnancy preferences (DAP score range: 0.4–4.0; mean: 2.24, standard deviation: 1.02; Cronbach's α = 0.92). 40% used contraception at last sex. Women with a greater preference to avoid pregnancy were marginally more likely to use contraception compared to those more open to pregnancy (odds ratio = 1.73; *p* = 0.09).
45	Postpartum Note Template Implementation Demonstrates Adherence to Recommended Counseling Guidelines	Grotell et al. (2021)	Resident OB/GYN clinics	Measure change in adherence with recommended postpartum counseling guidelines before versus after implementing a standardized note template	Compared visits prior to implementation of the template (*n* = 100) with visits post‐implementation (*n* = 100) and observed for documentation of counseling on Pap smear, birth spacing, breastfeeding, contraception, depression, gestational diabetes mellitus, pre‐eclampsia, and sleep/fatigue.	In visits that occurred without use of the template, counseling was charted as low as 1.0% for birth spacing to as high as 86.0% for contraception. With use of the template, counseling was charted as 100% in all visits for each of the recommended counseling guidelines.
46	Preconceptional Motivational Interviewing Interventions to Reduce Alcohol‐Exposed Pregnancy Risk	Ingersoll et al. (2013)	Community women at risk for alcohol‐exposed pregnancy (AEP) from two cities in central Virginia	Test a one‐session motivational AEP prevention intervention for community women and compare outcomes to previous RCTs	Participants at risk for AEP (*N* = 217) were randomized to motivational interviewing and assessment feedback (EARLY), informational video, or informational brochure. Outcomes were drinks per drinking day (DDD), ineffective contraception rate, and AEP risk at 3 and 6 months.	All interventions were associated with decreased DDD, ineffective contraception rate, and AEP risk. Participants who received EARLY had larger absolute risk reductions in ineffective contraception and AEP risk, but not DDD. One‐session motivational interviewing may provide a new option in the continuum of care.
47	Reducing Preconception Risks Among African American Women with Conversational Agent Technology	Jack et al. (2015)	Convenience sample of 100 women (18–34 years) who self‐identified as African‐American (AA)/ Black, from 20 states and the District of Columbia	Assess the ability of a computerized conversational agent, “Gabby,” to mitigate preconception health (PCH) risks, particularly for African‐American and Black women	Women (*n* = 100) were screened for over 100 PCH risks and randomized to Gabby or control group. The Gabby group interacted with the system for up to 6 months; the control group received a letter indicating their health risks with a recommendation to talk with their clinician. The numbers, proportions, and types of risks were compared between the groups.	The Gabby group had greater reductions in the number (8.3 vs. 5.5 risks, *p* < 0.05) and the proportion (27.8% vs. 20.5%, *p* < 0.01) of risks compared to controls. Seventy‐eight percent reported that it “was easy to talk to Gabby” and 64% used information from Gabby to improve their health.
48	Improving the Health of Young African American Women in the Preconception Period Using Health Information Technology: A Randomized Controlled Trial	Jack et al. (2020)	National sample of 528 African American/Black women aged 18–34 years	Assess the impact of an online conversational agent, “Gabby,” on preconception risks among African‐American and Black women	Intervention participants received 12 months of tailored health advice and motivational interviewing. The control group received a letter listing their preconception risks and encouraging them to talk with a clinician. The primary outcome was stage of change at months 6 and 12.	Participants identified a mean of 21 preconception risks per woman (SD 9·9). At 6 months, intervention women reached the action or maintenance stage of change for 50.0% of those risks, compared with 42.7% in the control group (incidence rate ratio 1·16, 95% CI 1·07–1·26). This result persisted at 12 months.
49	Prospective Assessment of Pregnancy Intentions Using a Single‐ versus a Multi‐Item Measure	Kavanaugh & Schwarz (2009)	Family planning clinics in Pittsburgh	Develop a tool that captures the complexity of people's intentions to become pregnant	Women (*n* = 249) awaiting pregnancy test results at family planning clinics completed a survey containing both single‐ and multi‐item measures of pregnancy intentions. Chi‐square analyses were used to assess differences between subgroups of women.	The two measures showed 68% concordance. The single item categorized more women as ambivalent than did the multi‐item measure (58% vs. 44%).
50	Identifying and Addressing Gaps in Reproductive Health Education for Adolescent Girls with Type 1 Diabetes	Kohn et al. (2018)	Large pediatric endocrinology clinic in an academic medical center	Assess attitudes and behaviors regarding reproductive health education (RHE) in diabetes health care professionals and in adolescent girls with diabetes, and pilot the American diabetes association “Diabetes and Reproductive Health for Girls” booklet	Researchers surveyed 29 providers and 50 adolescent girls with type 1 diabetes about RHE experiences, attitudes, and behaviors. The RHE intervention was piloted with nine adolescent–parent dyads.	Adolescent girls with diabetes rarely receive education on pregnancy and contraception due to provider discomfort, limited knowledge, and limited time. RHE using easily‐accessible materials with an educator may help address this gap in care.
51	Routine Screening for Pregnancy Intention to Address Unmet Reproductive Health Needs in Two Urban Federally Qualified Health Centers	Kvach et al. (2017)	Two urban federally qualified health centers (FQHCs)	Measure change in rates of screening for pregnancy intention in primary care after a quality improvement project implementing One Key Question in the electronic medical record (EMR)	Measured change in screening rates for pregnancy intention before versus after quality improvement project. Responses were recorded in the EMR.	Screening rates of eligible women increased from 0% to 68.3% and from 49.0% to 80.3% at Sites 1 and 2 respectively. Adolescents were screened at significantly lower rates than adults. There was no significant difference in screening rates between English and non‐English speaking patients.
52	The Family Planning Quotient and Reproductive Life Index (FPQ/RepLI) Tool: A Solution for Family Planning, Reproductive Life Planning and Contraception Counseling	Madrigal et al. (2019)	Urban, public hospital in Chicago	Measure the proportion of women who indicated that the tool was helpful and that they would use it to track their reproductive goals	Cross‐sectional evaluation of the FPQ/RepLI tool. Patients and providers completed an evaluation survey rating their satisfaction with the tool. Survey responses were summarized using frequencies and percentages.	Most patients (*n* = 725, 91.9%) agreed that the tool was helpful and that they would use it to track their reproductive goals. Fifty‐five (83.5%) providers agreed that there is a need for reproductive health tools in clinical practice. Most agreed that the tool helped the patient communicate goals, aided in educating about contraception, and facilitated the discussion and decision making process about available contraceptives.
53	A Checklist to Assess Childbearing Intentions and Promote Referral to Preconception Care or Contraception: A Multi‐Site Study	Mirabal‐Beltran et al. (2021)	HIV providers in seven different cities	Assess HIV provider views on the value of a checklist designed to assess patients' preconception care (PCC) needs and guide implementation of PCC	HIV providers (*n* = 92) shared perspectives regarding a checklist to facilitate communication and referrals for preconception care. A sub‐sample (*n* = 27) shared feedback on a checklist designed for this purpose. Feedback was analyzed using a grounded theory approach to examine patterns and emergent themes.	Checklist benefits included standardization of care, assisting new/inexperienced providers, educational resource for patients, and aid in normalizing childbearing. Providers suggested checklists be simple, incorporated into the electronic medical record, and accompanied with appropriate referral systems.
54	Use of a Modified Reproductive Life Plan to Improve Awareness of Preconception Health in Women with Chronic Disease	Mittal et al. (2014)	San Francisco General Hospital Family Health Center	Evaluate a reproductive life plan designed to improve the knowledge of preconception and contraception health in women with chronic diseases	English‐and Spanish‐speaking women aged 18–40 years with diabetes, hypertension, or obesity were recruited. Using a revised reproductive life plan specific to these diseases, physicians provided reproductive plan counseling. Pre‐ and post‐counseling surveys were administered to patients on chronic disease and the effects on a potential pregnancy.	Significant increases were reported in understanding risks of pregnancy associated with diabetes, hypertension, and obesity. After counseling, women increased their knowledge about a reproductive plan and increased support and information to make reproductive health choices.
55	Development and Psychometric Testing of the Attitude Toward Potential Pregnancy Scale	Paterno et al. (2014)	Two OB/GYN clinics and one family planning clinic in Baltimore, Maryland	Develop and test a comprehensive tool for measuring women's attitudes toward the possibility of becoming pregnant	Participants completed a computer‐based survey as part of a larger retrospective mixed‐methods study. The attitude toward potential pregnancy scale was assessed using exploratory factor analysis and hypothesis testing.	All items in the scale loaded to a single factor. Internal consistency was demonstrated with a Cronbach's alpha of 0.86. The odds of using highly effective contraception decreased 8% with each point increase in score, supporting construct validity.
56	Interconception Care for Mothers During Well‐Child Visits With Family Physicians: An IMPLICIT Network Study	Rosener et al. (2016)	Twelve US family medicine academic practices participating in the IMPLICIT network	Investigate interconception care practices by family physicians at well‐child visits (WCV), with focus on maternal depression, tobacco use, folic acid supplementation, and family planning	A convenience sample of mothers completed a survey about well‐child visits. Maternal history, behaviors, and the frequency of the child's physician addressing maternal depression, tobacco use, family planning, and folic acid were assessed, along with receptivity to advice.	Family physicians provided key elements of interconception care at well‐child visits, and mothers were highly receptive to advice from their child's physician even if they received primary care elsewhere.
57	Pregnancy Preferences and Contraceptive Use Among US Women	Samari et al. (2020)	Reproductive health and primary health facilities in Arizona, New Jersey, New Mexico, South Carolina, Texas	Understand the relationship between individuals' pregnancy preferences and contraceptive use using the Desire to Avoid Pregnancy (DAP) scale	Researchers used multivariable logistic, multinomial logistic, and linear regression models to examine the associations among DAP scores (range: 0–4) and contraceptive use and identify factors associated with discordance between DAP and use of contraception.	Participants with a greater preference to avoid pregnancy had higher odds of contraceptive use (aOR = 1.63, 95% CI: 1.31, 2.04) and used contraceptives more consistently. However, higher preference to avoid pregnancy was not associated with type of contraceptive method used.
58	Clinical Decision Support to Promote Safe Prescribing to Women of Reproductive Age: A Cluster‐Randomized Trial	Schwarz et al. (2012)	One academic and one community‐based practice	Evaluate whether computerized clinical decision support (CDS) can increase primary care providers' (PCPs') provision of family planning services when prescribing potentially teratogenic medications to women who can become pregnant	PCPs (*n* = 41) were randomized to receive one of two types of CDS when ordering potentially teratogenic medications: ‘the simple’ CDS provided a cautionary alert; the ‘multifaceted’ CDS provided tailored information and a structured order set. Change in documented family planning services was assessed and PCPs completed pre and post‐implementation surveys.	Both systems were associated with slight increases in provision of family planning services, without a significant difference by CDS complexity. PCPs improved significantly in several counseling and prescribing practices. The multifaceted group reported a greater increase in the number of times per month they discussed the risks of medication use during pregnancy (multifaceted: +4.9 ± 7.0 vs. simple: +0.8 ± 3.2, *p* = 0.03).
59	Counseling About Medication‐Induced Birth Defects with Clinical Decision Support in Primary Care	Schwarz et al. (2013)	Three community‐based family practice clinics or an academic general internal medicine clinic	Evaluate how computerized clinical decision support (CDS) affects the counseling women receive when primary care providers (PCPs) prescribe potential teratogens and how this counseling affects women's behavior	Women visiting a participating clinic were invited to complete a survey 5–30 days after their visit. Women who received prescriptions were asked if they were counseled about teratogenic risks or contraception and if they used contraception at last intercourse.	With or without CDS, women prescribed potential teratogens were more likely than women prescribed safer medications to receive counseling. Women who were pregnant or trying to conceive were not more likely to report counseling. Women who received counseling were more likely to use contraception.
60	Promoting Safe Prescribing in Primary Care With a Contraceptive Vital Sign: A Cluster‐Randomized Controlled Trial	Schwarz et al. (2012)	Primary care internal medicine clinic at University of Pittsburgh	Measure change in contraceptive documentation and provide with a tool designed to help primary care physicians identify patients who need preconception or contraceptive counseling, particularly when teratogenic medications are prescribed	Internists in the intervention group (*n* = 26) were provided with information on their female patients' pregnancy intentions and contraceptive use immediately before visits; internists in the control group (*n* = 27) received only standard intake information. Data were abstracted from the electronic health record for 5371 visits by 2304 women aged 18–50 years.	Documentation of contraception increased from baseline, from 23% to 57% in the intervention group, but remained 28% in the control group. For visits involving a teratogenic prescription, documentation increased from 14% to 48% in the intervention group and decreased from 29% to 26% in the control group. However, provision of new family planning services increased only minimally with this intervention.
61	Computerized Counseling for Folate Knowledge and Use: A Randomized Controlled Trial	Schwarz et al. (2008)	Two urgent care clinics in San Francisco: one academic and one with a county hospital	Evaluate whether computer‐assisted counseling and the provision of free folate tablets increases women's knowledge and use of folate supplements	Participants received a 15‐min computerized educational session and 200 folate tablets. The primary outcome was the knowledge that folate can prevent birth defects; secondary outcomes included the self‐reported use of a folate supplement at follow‐up.	A one‐time, brief, computerized counseling session about folate with the provision of free folate tablets increased the knowledge and use of folate supplements among women ≥6 months later.
62	Feasibility Study of Family Planning Services Screening as Clinical Decision Support at an Urban Federally Qualified Health Center Network	Shah et al. (2019)	Federally qualified health center (FQHC) in New York	Assess the feasibility of an intervention introducing family planning services (FPS) screening clinical decision support to improve provision of contraception and/or preconception services for reproductive age women	An FPS screening prompt was implemented for support staff to ask women ages 13–44. The response was displayed in the electronic medical record for the provider to review, linked to a documentation tool. Staff comfort with the screening was evaluated before and after implementation. Feasibility was assessed by screening rate and documentation of family planning services.	This study demonstrated high staff acceptability of the intervention at all sites and a high screening rate with a significant increase in family planning documentation rate at the pilot site during the intervention period.
63	Integration of Family Planning Services into a Sexually Transmitted Disease Clinic Setting	Shlay et al. (2013)	Denver Metro Health Clinic, Denver Public Health STD clinic	Assess an electronic eligibility reminder for family planning services (FPS) during an STD clinic visit; measure FPS use, additional cost of integrated services, and patient/provider satisfaction; explore the impact on incident pregnancy and STDs	Quasi‐experimental design compared enrollment and patient/provider satisfaction before and after implementation. Incident pregnancy and STD 12 months after the initial visit before and after were explored. Time and cost were calculated.	An electronic eligibility reminder of FPS increased FPS use. Integration of FPS with STD services is feasible, is well‐accepted, and increases costs minimally. Integration may reduce pregnancy rates without increasing STD rates.
64	Effects of Clinic‐Level Implementation of One Key Question on Reproductive Health Counseling and Patient Satisfaction	Song et al. (2021)	Two primary care and two OB/GYN practices in suburbs near Chicago	Evaluate the effect of clinic‐level implementation of the One Key Question (OKQ) intervention, including physician and staff training and workflow adjustments, on reproductive counseling and patient satisfaction	Cross‐sectional survey of patients before and after implementation of OKQ at intervention practices, and in same time period at control practices providing usual care. Chi square tests were used to assess OKQ's effects on counseling rates and patient satisfaction, comparing intervention to control practices across time points.	Practices implementing OKQ showed significant increases in patient satisfaction while usual care satisfaction declined in the same time period. Receipt of reproductive counseling increased in intervention practices but the difference with control practices was non‐significant.
65	Corrigendum to "Effects of Clinic‐level Implementation of One Key Question on Reproductive Health Counseling and Patient Satisfaction”	Song et al. (2021)		Correct prior publication (citation #64)		The authors made corrections to the sub‐heads of Tables 1 and 3. This did not change the study's results.
66	Effectiveness of Clinical Decision Support to Enhance Delivery of Family Planning Services in Primary Care Settings	Srinivasulu et al. (2020)	FQHC network in New York	Estimate the effect of clinical decision support (CDS) on delivery of family planning, including preconception and contraception services, in primary care	Used difference‐in‐differences design to at the visit‐level, measuring documentation of family planning services before and after implementation. Logistic regression with a sample subset was used to evaluate intervention effect on the patient‐level.	This clinical decision support modestly improved documentation of family planning services in this primary care network; effect varied across sites.
67	Increase in Contraceptive Counseling by Primary Care Clinicians After Implementation of One Key Question at an Urban Community Health Center	Stulberg et al. (2019)	Urban community health center in Chicago	Assess if implementing One Key Question in the Electronic Medical Record (EMR), coupled with brief clinician training, would increase rates of contraceptive and preconception counseling	Female patients, ages 18–49, were surveyed after their visit to compare pre‐ versus post‐intervention rates of contraceptive and preconception counseling.	Patients reported significantly higher rates of their clinician counseling them about contraception (52% vs. 76%, *p* = 0.040) and recommending a long‐acting reversible contraceptive (LARC) method (10% vs. 32%, *p* = 0.035). There were no significant changes in preconception counseling.
68	One Key Question and the Desire to Avoid Pregnancy Scale: A Comparison of Two Approaches to Asking About Pregnancy Preferences	Stulberg et al. (2020)	Primary care and OB/GYN practices	Facilitate assessment of patients' pregnancy preferences by comparing responses to OKQ with the Desire to Avoid Pregnancy (DAP) scale and assess their relationships to patient‐reported reproductive health behaviors	In after visit surveys, women ages 18–49 (*n* = 177) answered “Would you like to become pregnant in the next year?” and the 14‐item DAP scale. Logistic regression was used to test the association of OKQ and DAP with contraceptive and folic acid use.	One Key Question responses correlate with DAP scores, and contraceptive use correlates with not desiring pregnancy by both approaches.
69	Quantitative and Qualitative Impact of One Key Question on Primary Care Providers' Contraceptive Counseling at Routine Preventive Health Visits	Thorman et al. (2022)	Eight FQHCs in Utah	Describe the effect of OKQ implementation on contraceptive counseling rates at preventive health visits and evaluate primary care providers' perception of OKQ implementation on their contraceptive counseling practices	Implementation included a brief training and inclusion of OKQ in the EMR. Changes in screening and contraceptive counseling documentation rates were assessed using interrupted‐time‐series analysis. Semi‐structured providers were interviewed and analyzed thematically to create an explanatory framework.	OKQ did not change documented rates of contraceptive counseling and uptake was low in quantitative and qualitative analyses.
70	Cluster Randomized Trial of a Pre/Interconception Health Intervention for Mothers in Pediatric Visits	Upadhya et al. (2020)	Pediatric primary care practices	Assess the effectiveness of a pre/interconception women's health intervention delivered during pediatric primary care using a cluster randomized trial	Mothers in the intervention group completed a pre/interconception health screening tool and discussed results with their child's clinician. Mothers in the comparison group did not receive either. All received written preconception health information and a 90‐day supply of multivitamins. Primary outcomes at 6 and 12 months included contraception use, pregnancy, and access to and use of preventive health care. Secondary outcomes included daily folic acid supplementation, smoking, and substance use.	There was no significant effect of the intervention on contraceptive use, pregnancy incidence, or use of preventive care. Assignment to the intervention increased the odds of daily folic acid use (odds ratio 1.82, 95% confidence interval 1.25, 2.63) during follow‐up. Intervention mothers were less likely to smoke at 6, but not 12 months.
71	A Preconception Care Program for Women in a College Setting	Wade et al. (2012)	College campus	Assess change in preconception health knowledge following a peer‐led educational program piloted by sophomore nursing students (*n* = 52)	Materials included a brochure on preconception health, a risk assessment tool, a video with stories of unplanned pregnancies, and a reproductive life plan book. Peer educators administered a pretest, guided discussions, assessed each woman's health risks and administered a posttest.	Following the preconception program with peer educators, posttest scores indicated significantly increased knowledge of preconception health.

Abbreviations: AA, African American; CDS, Clinical Decision Support; DAP, Desire to Avoid Pregnancy; EMR, Electronic Medical Record; FQHC, Federally Qualified Health Center; FP, Family Planning; IMPLICIT, Interventions to Minimize Preterm and Low Birth Weight Infants through Continuous Improvement Techniques; MVI, Multi Vitamin; OB/GYN, Obstetrics/Gynecology; OKQ, One Key Question; PATH, Pregnancy Attitudes, Timing and How important; PCC, Preconception Care; PCH, Preconception Health; PCP, Primary Care Provider; READY‐Girls, Reproductive‐health Education and Awareness of Diabetes in Youth for Girls; RH, Reproductive Health; RH‐SAT, Reproductive Health Self‐Assessment Tool; RLP, Reproductive Life Plan; STD, Sexually Transmitted Disease; WBV, Well Baby Visit; WCV, Well Child Visit; WIC, Women Infants and Children.

**TABLE 3:** Specific Tools and Standardized Approaches

Standardized tool/Approach	Description	Setting(s) tested	Summary of findings	Quality of Research	Studies
Specific tools					
Attitude Toward Potential Pregnancy Scale (APPS)	5‐item survey that captures a variety of aspects of pregnancy attitudes and generates a score where higher scores correspond to more positive pregnancy attitude	2 OB/GYN clinics and one family planning clinic in Baltimore, Maryland	The APPS may be a useful tool for understanding pregnancy attitude in clinical practice.	Acceptability study/validated measure	Paterno & Han, 2014
Contraceptive vital sign	Information on women's pregnancy intentions and contraceptive use is collected as a “contraceptive vital sign” for primary care providers to reference immediately before visits	Primary care clinic at University of Pittsburgh	A contraceptive vital sign improves documentation of contraceptive use; however, ongoing efforts are needed to improve provision of preconception and contraceptive services.	Tested with RCT	Schwarz et al. 2012
Desire to Avoid Pregnancy (DAP)	14‐item scale that measures the ranges of women's preferences regarding a potential future pregnancy, with items covering three domains: desires, emotions, and perceived consequences	Seven reproductive health and primary health care facilities in Arizona, New Jersey, New Mexico, South Carolina, and Texas (Samari et al. 2020), methadone clinics in Los Angeles (Gipson et al. 2021)	Women with greater preference to avoid pregnancy had higher odds of contraceptive use; however, higher preference to avoid pregnancy was not associated with more effective contraceptive methods.	Observational study with outcome measure	Stulberg et al. 2020, Gipson et al. 2021, Samari et al. 2020
Family Planning Quotient (FPQ) and Reproductive Life Index (RepLI) (FPQ/RepLI)	A visual tool demonstrating a woman's reproductive goals that providers can use to facilitate goal‐oriented management	Urban, public hospital in Chicago	Most agreed that the tool helped the patient communicate goals, aided in educating about contraception, and facilitated the discussion and decision making process about available contraceptives. The tool gives patients a resource for family and reproductive goal planning. Broad dissemination among other medical specialties beyond obstetrics and gynecology may make reproductive life planning accessible to more women.	Observational study with outcome measure	Madrigal et al. 2019
Gabby	A Web‐based virtual animated health counselor	National sample of 528 Black or African‐American women aged 18–34 years	Web‐based conversational agents like Gabby are associated with preconception risk reduction and are a viable medium for delivering longitudinal preconception care counseling to adolescents and young adults.	Tested with RCT	Jack et al. 2015, Jack et al. 2020, Gardiner et al. 2020, Bickmore et al. 2020
IMPLICIT ICC questionnaire	The IMPLICIT ICC model includes screening and brief intervention for mothers at well‐child visits for smoking, depression, multivitamin use, and family planning	Well‐child visits at family medicine clinical sites participating in the IMPLICIT (Interventions to Minimize Preterm and Low Birth Weight Infants Through Continuous Improvement Techniques) network	Mothers who received an intervention were more likely to report taking an multivitamin at the subsequent well‐child visit and were also more likely to report discussions with their child's doctor for family planning, depression screening, and folic acid supplements.	Observational study with outcome measure	DeMarco et al. 2021, Frayne et al. 2021, Rosener et al. 2016
MyFamilyPlan	A web‐based preconception health education module	Well‐woman visits at an urban academic medical center in California	MyFamilyPlan exposure was associated with a significant increase in the proportion of women who reported discussing reproductive health with providers and may promote preconception health awareness.	Tested with RCT	Batra et al. 2018
MyPath	Web‐based decision support tool integrating reproductive goals assessment, information about optimizing health before pregnancy, and contraceptive decision support	Primary care centers in the veterans administration	MyPath was highly acceptable to women, increased the proportion of primary care visits addressing reproductive needs, and improved decision quality without increasing providers' perceived workload.	Tested with RCT	Callegari et al. 2021
Pregnancy Attitudes, Timing, and How important is pregnancy prevention (PATH)	A three‐question approach that seeks to capture patients' emotional orientation toward pregnancy: “How would you feel about getting pregnant in the next month, with response options ranging from 0 with the anchor of ‘worst feeling you can imagine’ to 100 with the anchor of ‘happiest you could possibly feel.’” “What are your future pregnancy plans?” “How important is it to you to not get pregnant until you are ready?” (for those who intend to become pregnant at some point in the future) and “How important is it to you to not get pregnant now or in the future?”	Family planning health centers in Salt Lake City, Utah	Pregnancy attitudes, plans and emotions inform clients' contraceptive needs and behaviors. Client‐centered contraceptive care may benefit from a more nuanced PATH approach rather than relying on a single time‐oriented question about pregnancy intention.	Observational study with outcome measure	Geist et al. 2019
One Key Question (OKQ)	Prompts physicians to provide appropriate counseling to each patient via the question, “Do you intend to become pregnant in the next year?”	Urban community health center in Chicago (Stulberg et al. 2019), primary care and OB/GYN practices (Song et al. 2021), FQHCs in Utah (Thorman et al. 2022), a VA in Utah (Gawron et al. 2021), one OBGYN and family medicine clinic (Ferketa et al. 2022), two urban FQHCs (Kvach et al. 2017)	One Key Question (OKQ) was generally found to increase rates of preconception screening. OKQ had minimal impact on clinical workflow during its implementation.	Tested with RCT	Stulberg et al. 2019, song et al. 2021, Thorman et al. 2022, Gawron et al. 2021, Ferrketa et al. 2022, Kvach et al. 2017
READY‐Girls	Preconception counseling program for teens with type 1 diabetes involving: viewing two CD‐ROMs, reading a book version, and having a brief research nurse counseling session during three consecutive diabetes clinical visits over a 9‐month period Separate arm: mothers were also assessed for their knowledge of diabetes and pregnancy and given the READY‐Girls book intervention	Diabetes clinics at university hospitals	READY‐Girls appeared to have long‐term sustaining effects on PC knowledge, beliefs, and intentions to initiate discussion with health care providers that could improve reproductive health behaviors and outcomes. Additionally, mothers can play a vital role in initiating discussions regarding reproductive health with their daughters and reinforcing preconception counseling.	Tested with RCT	Kohn et al. 2018, Charron‐Prochownik et al. 2008, Fisschl et al. 2010, Chraron‐Prochownik et al. 2013, Charron‐Prochownik et al. 2014
REFRAMED PLUS	A multi‐pronged peer education preconception health program that uses the mnemonic REFRAMED PLUS (Reproductive awareness, Environmental toxins and teratogens, Folic acid, Review of genetic history, Alcohol, tobacco, and other substance abuse, Medical care and medications, Evaluate immunizations and infectious disease risk, Domestic violence and psychosocial issues, Pregnancy, Labor, Understanding, Self‐empowerment) to address eight preconception risk areas and reproductive life planning	College campuses	Following the preconception program, post‐est scores indicated increased knowledge of preconception health. For preconception health care to be successful, preconception risk assessments, education and counseling must be addressed by nurses every time a young woman receives care	Observational study with outcome measure	Wade et al. 2012
Reproductive Health Self Assessment Tool (RH‐SAT)	A four‐part questionnaire that asks women to consider their feelings about pregnancy and provides information on reproductive health topics	Urban community health center and federally qualified health center in Chicago	Participants felt that the RH‐SAT provided new information and would prompt them to discuss contraception and/or preparing for pregnancy with their clinician. This tool has the potential to facilitate patient‐clinician discussion of reproductive health in primary care.	Observational study with outcome measure	Bello et al. 2013, Bello et al. 2020
Reproductive Health Attitudes and Behavior (RHAB)	48‐item instrument for preconception planning of young women with diabetes	Four major university‐based medical centers with pediatric diabetes clinics, located in Pittsburgh, PA; St. Louis, MO; Boston, MA; Detroit, MI	RHAB appears to have acceptable levels of validity/reliability for use with female adolescents with diabetes.	Acceptability study/validated measure	Charron‐Prochownik et al. 2006
Reproductive Health Service Needs Question	“Can I help you with any reproductive health services today, such as birth control or planning for a healthy pregnancy?”	Primary care settings in New York state (urban, suburban, and rural)	Participants had the most positive response to the proposed question “Can I help you with any reproductive health services today, such as birth control or planning for a healthy pregnancy?” based on its open‐endedness, inclusiveness, and promotion of reproductive autonomy.	Acceptability study/validated measure	Manze et al. 2020
Reproductive Life Plan Tool (RLPT)	A tool that prompts physicians to ask general questions about women's contraceptive needs and offer referral services for mothers who desire contraception services	Well‐baby visits at an academic center	Findings indicate that use of the Reproductive Life Plan Tool is generally feasible during routine infant care and acceptable to pediatric resident physicians with recognition of challenges to implementation.	Observational study with outcome measure	Caskey et al. 2016
Women's Health Screening Tool	15 item “Healthy Moms, Healthy Babies” survey that addresses different aspects of pre/interconception care	Pediatric primary care practices (well‐child visits)	Pediatric visits are an opportune location for addressing maternal health and this intervention demonstrated feasibility and improved outcomes for some but not all outcomes. Attention to maternal health needs in pediatric visits during infancy may be important for maintaining positive pre/interconception health behaviors.	Tested with RCT	Upadhya et al. 2020
Other standardized approaches					
Checklists					
Checklist to assess preconception care needs of patients living with HIV	Two‐page checklist that collects information from patients living with HIV on one side and an algorithm for providers to follow on the second	HIV primary care clinics in seven different cities	Findings support a need for a checklist tool to assist in conversations about reproductive intentions/desires. Additional referral or innovative consultative services will be needed as more persons living with HIV/AIDS are engaged on the topic of childbearing.	Acceptability study/validated measure	Mirabal‐Beltran et al. 2021
Preconception care chart insert and provider education	Two‐part intervention^1^: a 1‐h lecture for all providers and^2^ a standardized checklist format encompassing all areas of preconception care, including reproductive, medical, social and family histories, nutrition, medication use, and family planning	Outpatient gynecology clinic at an inner‐city hospital in New York	The combination of education about preconception care and insertion of a standardized form into a patient's chart led to a clear improvement in the documentation of preconception care.	Observational study with outcome measure	Bernstein et al. 2000
Counseling					
Computer‐assisted counseling	One‐time, brief, computerized counseling session about folate with the provision of free folate tablets	Two urgent care clinics in San Francisco, one affiliated with an academic center and one with a county hospital	A one‐time computerized counseling session about folate with the provision of free folate tablets increased the knowledge and use of folate supplements among women ≥6 months later.	Tested with RCT	Schwarz et al. 2008
Preconception health screening and counseling	15‐item preconception health screening and counseling	Four urban pediatric practices in Baltimore, Maryland	Offering vitamins and recommending folate intake to mothers within pediatric practice can increase use. Pediatric practice is an important contact point and context for improving maternal folate use.	Tested with RCT	Chilukuri et al. 2018
Preconception risk assessment and brief counseling	Reproductive health risk assessment questionnaire followed by 10–15 min counseling	A Women, Infants, and Children (WIC) clinic in Clayton County, Georgia	Women, Infants, and Children clinics constitute a suitable location for identifying low‐income women in need of preconception and reproductive health services and at risk for poor reproductive health outcomes.	Observational study with outcome measure	Dunlop et al. 2013, Dunlop et al. 2013
Electronic Health Record (EHR) additions					
Computerized clinical decision support (CDS)	CDS which alerts providers to risks of medication‐induced birth defects when ordering potentially teratogenic medications for women who may become pregnant	Academic internal medicine clinics and community‐based family practice clinics (Schwarz et al. 2012, Schwarz et al. 2013), family medicine staffed primary care network (Srinivasulu et al. 2020)	CDS systems hold promise for increasing provision of family planning services when fertile women are prescribed potentially teratogenic medications.	Tested with RCT	Schwarz et al. 2012, Schwarz et al. 2013, Srinivasulu et al. 2020
Electronic eligibility reminder for provision of family planning services	Family planning services (FPS) screening prompt on electronic health records that identifies populations that may benefit from FPS	Denver Metro Health Clinic (Shlay et al. 2013), federally qualified health centers (Shah et al. 2019)	An electronic eligibility reminder of FPS increased FPS use. Integration of FPS with STD services is feasible, is well‐accepted, and increases costs minimally. The FPS screening decision intervention is also feasible in an FQHC setting.	Observational study with outcome measure	Shlay et al. 2013, Shah et al. 2019
Electronic Health Record Template	Electronic health record (EHR) template for postpartum counseling	Resident‐run OB/GYN clinic	An EHR template for documentation of postpartum visits is associated with resident adherence with recommended postpartum counseling guidelines. Managers in hospitals and clinical practices should consider incorporating OBGYN‐specific EHR note templates to improve quality and increase adherence with recommended guidelines during postpartum visits.	Observational study with outcome measure	Grotell et al. 2021
Motivational interviewing					
Computer‐Assisted Motivational Interviewing (CAMI)	Based on teens' responses to current sexual relationships and contraceptive and condom use intentions, CAMI algorithms produced a summary depicting whether teens were at no, low, medium, or high risk for pregnancy and STIs. Interventionists recruited from the community then conducted a 20‐min motivational interviewing session to enhance the teen's motivation to use contraception and remain nonpregnant.	Urban prenatal clinics in Baltimore, Maryland serving low‐income, predominantly Black and African‐American communities	Receipt of two or more CAMI sessions, either alone or within a multicomponent home‐based intervention, reduced the risk of rapid subsequent birth to adolescent mothers.	Tested with RCT	Barnet et al. 2009
One‐session motivational alcohol‐exposed pregnancy prevention intervention	One‐session alcohol‐exposed pregnancy prevention intervention	Two cities in central Virginia	The one‐session EARLY intervention had less powerful effects than multi‐session alcohol‐exposed pregnancy prevention interventions among community women, but may provide a new option in a continuum of preventive care.	Tested with RCT	Ingersoll et al. 2013
Reproductive life plan questionnaires					
Reproductive life plan for women with chronic diseases	A multi‐page survey that helps patients set pregnancy goals based on their pre‐existing pregnancy risk factors and chronic conditions	San Francisco General Hospital Family Health Center	A reproductive life plan is a brief, cost‐effective preconception, and contraception counseling tool in the primary care setting for women with chronic diseases. This tool increases knowledge about reproductive health and enables women with chronic diseases to make informed decisions about their reproductive future.	Observational study with outcome measure	Mittal et al. 2014
Reproductive life plan to identify women using teratogenic medications	A multi‐page questionnaire that assesses women's pregnancy intentions and pertinent health history for pregnancy risk factors	Toledo‐Lucas County Healthy Start Program	The reproductive life plan can be a useful tool to identify women of childbearing age who require intervention due to use of potentially teratogenic medications. Efforts are needed to ensure complete and accurate reporting of medication use in reproductive life plans, and to promote effective contraceptive use among women taking potentially teratogenic medications.	Observational study with outcome measure	DiPietro Mager et al. 2018
Reproductive life plan questionnaire	A questionnaire that assess participants' desire to have a child and their contraceptive practices; these responses were then attached to the medical record for provider's use	Publicly funded clinics	Primary care practices should consider implementing a reproductive plans assessment to facilitate linkage of patients to appropriate family planning, preconception, and sexually‐transmitted infection services.	Observational study with outcome measure	Dunlop et al. 2010
Pregnancy intention measure	A survey containing single‐ and multi‐item measures of pregnancy intentions	Family planning clinics in Pittsburgh	Prospective assessment of pregnancy intention with either a single‐ or a multi‐item measure may allow for a more nuanced assessment of pregnancy intention than current measures. The multi‐item measure may reduce the number of women categorized as ambivalent and aid the development of targeted contraceptive and preconception counseling interventions.	Observational study with outcome measure	Kavanaugh and Schwarz 2009

### 3.3 Results from the mixed methods appraisal tool quality assessment

Each included study collected data to address clearly defined research questions and therefore met the baseline criteria for assessing quality using the Mixed Methods Appraisal Tool. Assessment findings for each category of study are as follows: the qualitative research and mixed methods studies we assessed met 80% or more of the included criteria. Most randomized controlled trials reported complete outcome data, included groups that were comparable at baseline, and study participants adhered to the assigned intervention. Nine of 14 studies did not provide adequate information to determine whether outcome assessors were blinded to the intervention. All quantitative non‐randomized studies used appropriate measurements for the intervention and the majority reported complete outcome data. However, only 3/16 studies provided enough information to indicate that the study sample was representative of the target population. Overall, the quantitative descriptive studies utilized appropriate sampling strategies (11/11), measurements (10/11), and statistical analysis (10/11) but only one provided enough information to determine that the sample was representative of the target population.

### 3.4 Specific tools and other standardized approaches

We found 17 specific tools, and an additional five standardized approaches not associated with a specific tool. Many of the specific tools incorporated a combination of approaches. For example, the Contraceptive Vital Sign used a patient intake questionnaire along with a clinical decision support prompt in the clinic's electronic medical record. Other tools constituted a single approach, such as the Desire to Avoid Pregnancy scale questionnaire, or the Pregnancy Attitudes How Important Timing (PATH) counseling framework.

A number of these various tools and approaches have been tested in specific primary care settings such as Federally Qualified Health Centers (One Key Question, Reproductive Health Self Assessment Tool, electronic medical record reminder) and the Veterans Administration (My Path, One Key Question). Others were designed for specialty care outpatient settings, like READY‐Girls (Reproductive health Education and Awareness of Diabetes in Youth for Girls). Table 3 includes a comprehensive list of specific tools and standardized approaches, as well as all the settings in which they have been tested.

## 4 DISCUSSION

We identified 22 standardized tools and approaches for preconception, interconception, or pregnancy intention screening in U.S. medical care settings. Some approaches have been studied with randomized controlled trials, including two web‐based tools (MyFamilyPlan and Gabby), one patient‐facing pre‐visit tool (MyPath), one in‐clinic screening tool (One Key Question), three standardized counseling approaches (READY‐Girls preconception counseling for patient with diabetes, Computer‐Assisted Motivational Interviewing, and a one‐session motivational interviewing on alcohol use), two electronic prompts (Contraceptive Vital Sign and a CDS focused on teratogen prescribing), and one maternal health screening embedded in the well child visit (Healthy Moms Healthy Babies). Others have been described in multiple observational studies with post‐implementation assessments of outcomes, such as folic acid use (IMPLICIT) and family planning counseling (Family Planning Services prompt). Other approaches are based on sound theory and have data indicating that they are acceptable and appreciated by patients (Reproductive Health Self‐Assessment Tool, Reproductive Life Index, Reproductive Health Service need question) or are associated with contraceptive method selection (Pregnancy Attitudes, Timing, and How important is pregnancy prevention). And finally, some are validated research tools with potential application in clinical settings (Attitude Toward Potential Pregnancy Scale, Reproductive Health Attitudes and Behavior, checklist for preconception care needs of people living with HIV).

For specific health conditions and behaviors, where there is strong evidence that providing preconception care improves pregnancy outcomes, such as preconception glycemic control in patients with diabetes reducing the risk of major and minor congenital anomalies, and preconception use of folic acid reducing the risk of neural tube defects and related anomalies,^18^, ^19^ it is important to use validated tools and approaches found to be effective in clinical practice. Interventions such as READY‐Girls for adolescents with diabetes, or the IMPLICIT model for embedding interconception care within well‐child visits, provide evidence‐based strategies for implementing these practices.

For many other approaches, the evidence indicates that implementing a standardized prompt or screening tool leads to higher rates of screening and counseling, but these have yet to demonstrate changes in patient behavior or clinical outcomes. Similar to our findings, prior systematic reviews have found insufficient evidence on the effectiveness of pregnancy intention screening to change patient outcomes, although with our updated review we add multiple recent studies that indicate pregnancy intention screening leads to changes in counseling rates or patient satisfaction.^20^ Future research will ideally follow patients longitudinally and track multiple outcomes, including prevention of undesired pregnancy, better pre‐pregnancy health, and improved perinatal outcomes.

Our approach had several limitations that clinicians should consider when applying these tools: First, we only reviewed studies in English that tested tools in the United States. It is possible that tools developed in other languages and countries could be adapted and prove clinically useful in the United States. Our strategy may have disproportionately excluded tools that would be useful in U.S.‐based patient populations for whom English is not the primary language. A second limitation is the exclusion of studies that only looked at contraceptive use (including method selection, continuation, etc.), unless it was clear the tool would also be applicable for preconception counseling. In applying this exclusion criterion, it is possible we inadvertently excluded tools that could be useful for interconception/preconception care.

Nonetheless, the findings of this systematic review are significant in summarizing a diverse and growing body of research: the implementation of clinical tools for preconception and interconception care, and pregnancy intention screening. Using our findings, clinicians will be able to identify applicable tools based on their practice setting and patient population and understand the existing research about each tool. Researchers wishing to build on existing evidence, and those interested in adapting known tools to new settings, may also find this review useful. Routine use of evidence‐based preconception tools has the potential to help patients control the timing of conditions of future pregnancies and improve perinatal outcomes – especially for those with pre‐pregnancy chronic conditions or other risk factors for adverse outcomes. To achieve this potential benefit, ongoing research is needed to further assess the use of tools in everyday clinical practice.

## CONFLICT OF INTEREST

The authors declare that there is no conflict of interest.

## Supporting information


**Table S1.** Search strategies for different databases.

**Table S2.** Results of quality appraisal of included studies using the Mixed Methods Appraisal Tool (MMAT).
