# Supplementary material for: Preconception, Interconception, and reproductive health screening tools: A systematic review
Source: Health Serv Res. 2023 Jan 6;58(2):458–88. doi: 10.1111/1475-6773.14123 (PMC10012234; doi:10.1111/1475-6773.14123)
Supplement: Supplementary file 1 — Table S1. Search strategies for different databases. [file HESR-58-458-s001.docx]

**Supplemental Table 1. Search strategies for different databases**

| PubMed | |
| --- | --- |
| Primary Search | (("preconception care"[MeSH Terms] OR ("preconception"[All Fields] AND "care"[All Fields]) OR "preconception care"[All Fields] OR "interconception care"[All Fields] OR ("family planning services"[MeSH Terms] OR "family planning services"[All Fields] OR "family planning"[All Fields]) OR "contraception"[MeSH Terms] OR "contraception"[All Fields] OR "contraceptives"[All Fields]) AND ("reproductive health services"[MeSH Terms] OR ("reproductive"[All Fields] AND "health"[All Fields] AND "services"[All Fields]) OR "reproductive health services"[All Fields] OR "counseling"[MeSH Terms] OR "counseling"[All Fields]) AND ("English"[Language] AND 2000/01/01:2022/03/01[Date - Publication])) NOT ("animals"[MeSH Terms] NOT "humans"[MeSH Terms]) |
| *Title Only* | "Scoping Review"[Title] OR "Systematic Review"[Title] |
| *Title Only* | ((((((((((((((((((((((((((((((((((((((((((((((((((((((((((((((((((((((((((((((((Afghanistan[Title]) OR (Argentina[Title])) OR (Armenia[Title])) OR (Australia[Title])) OR (Austria[Title])) OR (Bangladesh[Title])) OR (Belgium[Title])) OR (Botswana[Title])) OR (Brazil[Title])) OR (Burkina Faso[Title])) OR (China[Title])) OR (Congo[Title])) OR (Cambodia[Title])) OR (Cameroon[Title])) OR (Chile[Title])) OR (Colombia[Title])) OR (Cote Ivoire[Title])) OR (Czech Republic[Title])) OR (Denmark[Title])) OR (Egypt[Title])) OR (England[Title])) OR (Estonia[Title])) OR (Finland[Title])) OR (France[Title])) OR (Ethiopia[Title])) OR (Germany[Title])) OR (Ghana[Title])) OR (Greece[Title])) OR (Guinea[Title])) OR (Hungary[Title])) OR (Indonesia[Title])) OR (Iran[Title])) OR (Ireland[Title])) OR (Israel[Title])) OR (Italy[Title])) OR (Jamaica[Title])) OR (Japan[Title])) OR (Jordan[Title])) OR (Kenya[Title])) OR (Kuwait[Title])) OR (Lebanon[Title])) OR (Lithuania[Title])) OR (Malawi[Title])) OR (Malaysia[Title])) OR (Myanmar[Title])) OR (Mozambique[Title])) OR (Nepal[Title])) OR ("New Zealand"[Title])) OR (Netherlands[Title])) OR (Nigeria[Title])) OR (Pakistan[Title])) OR (Peru[Title])) OR (Philippines[Title])) OR (Poland[Title])) OR (Portugal[Title])) OR (Romania[Title])) OR (Russia[Title])) OR (Rwanda[Title])) OR (Saudi Arabia[Title])) OR (Scotland[Title])) OR (Senegal[Title])) OR (Serbia[Title])) OR (Sierra Leone[Title])) OR (Singapore[Title])) OR ("South Africa"[Title])) OR (Korea[Title])) OR (Spain[Title])) OR (Sri Lanka[Title])) OR (Sub Saharan[Title])) OR (Swaziland[Title])) OR (Sweden[Title])) OR (Switzerland[Title])) OR (Tanzania[Title])) OR (Thailand[Title])) OR (Turkey[Title])) OR (Uganda[Title])) OR (Uruguay[Title])) OR (Vietnam[Title])) OR (Wales[Title])) OR (Zambia[Title])) OR (Zimbabwe[Title]) |
| Web of Science | |
| Primary Search | ((TS=((preconception NEAR care) OR “preconception care” OR "interconception care" OR (interconception NEAR care) OR “family planning” OR contracept* )) AND TS=( (reproductive NEAR/10 health NEAR/10 services) OR “reproductive health services” OR counsel*)) NOT TS=((animals NOT humans)) |
| *Title Only Search* | (TI=(Scoping Review)) OR TI=(Systematic Review) |
| *Title Only Search* | (((((((((((((((((((((((((((((((((((((((((((((((((((((((((((((((((((((((((((((((((TI=(Afghanistan)) OR TI=(Argentina)) OR TI=(Armenia)) OR TI=(Australia)) OR TI=(Austria)) OR TI=(Bangladesh)) OR TI=(Belgium)) OR TI=(Botswana)) OR TI=(Brazil)) OR TI=(Burkina Faso)) OR TI=(China)) OR TI=(Congo)) OR TI=(Cambodia)) OR TI=(Cameroon)) OR TI=(Chile)) OR TI=(Colombia)) OR TI=(Cote Ivoire)) OR TI=(Czech Republic)) OR TI=(Denmark)) OR TI=(Egypt)) OR TI=(England)) OR TI=(Estonia)) OR TI=(Finland)) OR TI=(France)) OR TI=(Ethiopia)) OR TI=(Germany)) OR TI=(Ghana)) OR TI=(Greece)) OR TI=(Guinea)) OR TI=(Hungary)) OR TI=(India)) OR TI=(Indonesia)) OR TI=(Iran)) OR TI=(Ireland)) OR TI=(Israel)) OR TI=(Italy)) OR TI=(Jamaica)) OR TI=(Japan)) OR TI=(Jordan)) OR TI=(Kenya)) OR TI=(Kuwait)) OR TI=(Lebanon)) OR TI=(Lithuania)) OR TI=(Malawi)) OR TI=(Malaysia)) OR TI=(Myanmar)) OR TI=(Mozambique)) OR TI=(Nepal)) OR TI=(New Zealand)) OR TI=(Netherlands)) OR TI=(Nigeria)) OR TI=(Norway)) OR TI=(Pakistan)) OR TI=(Peru)) OR TI=(Philippines)) OR TI=(Poland)) OR TI=(Portugal)) OR TI=(Romania)) OR TI=(Russia)) OR TI=(Rwanda)) OR TI=(Saudi Arabia)) OR TI=(Scotland)) OR TI=(Senegal)) OR TI=(Serbia)) OR TI=(Sierra Leone)) OR TI=(South Africa)) OR TI=(Korea)) OR TI=(Sub Saharan)) OR TI=(Spain)) OR TI=(Sri Lanka)) OR TI=(Swaziland)) OR TI=(Sweden)) OR TI=(Switzerland)) OR TI=(Tanzania)) OR TI=(Thailand)) OR TI=(Turkey)) OR TI=(Uganda)) OR TI=(Uruguay)) OR TI=(Vietnam)) OR TI=(Wales)) OR TI=(Zambia)) OR TI=(Zimbabwe) |
| CINAHL | |
| Primary Search | ( ("preconception care" OR (MH "Prepregnancy Care") OR "interconception care" OR "family planning" OR (MH "Family Planning") OR contraception OR (MH "Contraception") ) AND ( reproductive health services OR (MH "Counseling") ) |
| *Title Only Search* | TI scoping review OR TI systematic review |
| *Title Only Search* | TI Afghanistan OR TI Argentina OR TI Armenia OR TI Australia OR TI Austria OR TI Bangladesh OR TI Belgium OR TI Botswana OR TI Brazil OR TI Burkina Faso OR TI China OR TI Congo OR TI Cambodia OR TI Cameroon OR TI Chile OR TI Colombia OR TI Cote Ivoire OR TI Czech Republic OR TI Denmark OR TI Egypt OR TI England OR TI Estonia OR TI Finland OR TI France OR TI Ethiopia OR TI Germany OR TI Ghana OR TI Greece OR TI Guinea OR TI Hungary OR TI India OR TI Indonesia OR TI Iran OR TI Ireland OR TI Israel OR TI Italy OR TI Jamaica OR TI Japan OR TI Jordan OR TI Kenya OR TI Kuwait OR TI Lebanon OR TI Lithuania OR TI Malawi OR TI Malaysia OR TI Myanmar OR TI Mozambique OR TI Nepal OR TI New Zealand OR TI Netherlands OR TI Nigeria OR TI Norway OR TI Pakistan OR TI Peru OR TI Philippines OR TI Poland OR TI Portugal OR TI Romania OR TI Russia OR TI Rwanda OR TI Saudi Arabia OR TI Scotland OR TI Senegal OR TI Serbia OR TI Sierra Leone OR TI South Africa OR TI Korea OR TI Sub Saharan OR TI Spain OR TI Sri Lanka OR TI Swaziland OR TI Sweden OR TI Switzerland OR TI Tanzania OR TI Thailand OR TI Turkey OR TI Uganda OR TI Uruguay OR TI Vietnam OR TI Wales OR TI Zambia OR TI Zimbabwe |

**The “title only” searches were run with the NOT operator to remove scoping reviews, systematic reviews, and country-specific studies outside the U.S.*

***PubMed and Web of Science uses the same time period: 1/1/2000 - 3/1/2022. CINAHL has different time period options and searches January 2000 - February 2022 (includes all February studies, stops at March).*
