# Supplementary material for: Preconception, Interconception, and reproductive health screening tools: A systematic review
Source: Health Serv Res. 2023 Jan 6;58(2):458–88. doi: 10.1111/1475-6773.14123 (PMC10012234; doi:10.1111/1475-6773.14123)
Supplement: Supplementary file 2 — Table S2. Results of quality appraisal of included studies using the Mixed Methods Appraisal Tool (MMAT). [file HESR-58-458-s002.docx]

**Supplemental Table 2. Results of quality appraisal of included studies using the Mixed Methods Appraisal Tool (MMAT)**

| **1. Qualitative Studies** | | | | | | |
| --- | --- | --- | --- | --- | --- | --- |
| **Author and Year** | **Citation Number** | **1.1 Is the qualitative approach appropriate to answer the research question?** | **1.2 Are the qualitative data collection methods adequate to address the research question?** | **1.3 Are the findings adequately derived from the data?** | **1.4 Is the interpretation of results sufficiently substantiated by data?** | **1.5 Is there coherence between qualitative data sources, collection, analysis and interpretation?** |
| Manze et al. (2020) | 9 | Yes | Yes | Yes | Yes | Yes |
| Bello et al. (2013) | 12 | Yes | Yes | Yes | Yes | Yes |
| Bello et al. (2020) | 23 | Yes | Yes | Yes | Yes | Yes |
| Mirabal-Beltran et al. (2021) | 53 | Yes | Yes | Yes | Yes | Yes |
| Ferketa et al. (2022) | 38 | Yes | Yes | Yes | Yes | Yes |
| **2. Quantitative Randomized Control Trial** | | | | | | |
| **Author and Year** | **Citation Number** | **2.1 Is randomization appropriately performed?** | **2.2 Are the groups comparable at baseline?** | **2.3 Are there complete outcome data?** | **2.4 Are outcome assessors blinded to the intervention provided?** | **2.5 Did the participants adhere to the assigned intervention?** |
| Song et al. (2021) | 64 | Yes | Yes | Yes | Can't tell | Yes |
| Chilukuri et al. (2018) | 32 | Yes | Yes | Yes | Can't tell | Can't tell |
| Fischl et al. (2010) | 39 | Can't tell | Yes | Yes | Can't tell | Yes |
| Charron-Prochownik et al. (2008) | 28 | Can’t tell | Can’t tell | Can’t tell | Can’t tell | Can’t tell |
| Charron-Prochownik et al. (2013) | 30 | Yes | Yes | Yes | Can't tell | Can't tell |
| Schwarz et al. (2008) | 61 | Yes | Can't tell | No | Yes | Yes |
| Jack et al. (2015) | 47 | Yes | Yes | Yes | Can't tell | Yes |
| Jack et al. (2020) | 48 | Yes | Yes | Yes | No | Yes |
| Gardiner et al. (2020) | 41 | Can't tell | Yes | No | Can't tell | Yes |
| Batra et al. (2018) | 22 | Yes | Yes | Yes | Yes | Yes |
| Schwarz et al. (2012) | 58 | Can't tell | Yes | Yes | Can't tell | Yes |
| Barnet et al. (2009) | 21 | Yes | Yes | Yes | Can't tell | Yes |
| Ingersoll et al. (2013) | 46 | Can't tell | Yes | No | Yes | Yes |
| Schwarz et al. (2012) | 60 | Can't tell | Yes | Yes | Can't tell | Yes |
| Upadhya et al. (2020) | 70 | Yes | Yes | Yes | Yes | Can't tell |
| **3. Quantitative Non-Randomized** | | | | | | |
| **Author and Year** | **Citation Number** | **3.1 Are the participants representative of the target population?** | **3.2 Are measurements appropriate regarding both the outcome and intervention (or exposure)?** | **3.3 Are there complete outcome data?** | **3.4 Are the confounders accounted for in the design and analysis?** | **3.5 During the study period, is the intervention administered (or exposure occurred) as intended?** |
| Shah et al. (2019) | 62 | Can't tell | Yes | Yes | No | Yes |
| Kvach et al. (2017) | 51 | Yes | Yes | Yes | No | Can't tell |
| Stulberg et al. (2019) | 67 | Yes | Yes | Yes | Yes | Can't tell |
| DeMarco et al. (2021) | 33 | Can't tell | Yes | Yes | Yes | Can't tell |
| Frayne et al. (2021) | 40 | Can't tell | Yes | Yes | No | Can't tell |
| Callegari et al. (2021) | 26 | Can't tell | Yes | Yes | No | Yes |
| Gawron et al. (2021) | 42 | Can't tell | Yes | Can't tell | No | Can't tell |
| Wade et al. (2012) | 71 | Can't tell | Yes | Can't tell | Can't tell | Yes |
| Bickmore et al. (2020) | 25 | Can't tell | Yes | No | Can't tell | Yes |
| Schwarz et al. (2013) | 59 | No | Yes | No | Yes | Can't tell |
| Srinivasulu et al. (2020) | 66 | Yes | Yes | Yes | Yes | Yes |
| Grotell et al. (2021) | 45 | Can't tell | Yes | Yes | No | Yes |
| Shlay et al. (2013) | 63 | Can't tell | Yes | Can't tell | Yes | Yes |
| Bernstein et al. (2000) | 24 | Can't tell | Yes | Yes | No | Can't tell |
| Dunlop et al. (2013) | 37 | Can't tell | Yes | Yes | No | Yes |
| Mittal et al. (2014) | 54 | Can't tell | Yes | Yes | Yes | Yes |
| **4. Quantitative Descriptive** | | | | | | |
| **Author and Year** | **Citation Number** | **4.1 Is the sampling strategy relevant to address the research question?** | **4.2 Is the sample representative of the target population?** | **4.3 Are the measurements appropriate?** | **4.4. Is the risk of nonresponse bias low?** | **4.5 Is the statistical analysis appropriate to answer the research question?** |
| Rosener et al. (2016) | 56 | Yes | Can't tell | Yes | Yes | Can't tell |
| Charron-Prochownik et al. (2006) | 31 | Yes | Can't tell | Yes | Can't tell | Yes |
| DiPietro et al. (2018) | 34 | Yes | Can't tell | Yes | Can't tell | Yes |
| Geist et al. (2019) | 43 | Yes | Can't tell | Yes | Can't tell | Yes |
| Gipson et al. (2021) | 44 | Yes | Can't tell | Yes | Can't tell | Yes |
| Kavanaugh & Schwarz (2009) | 49 | Yes | Yes | Yes | Can't tell | Yes |
| Kohn et al. (2018) | 50 | Yes | Can't tell | Yes | Can't tell | Yes |
| Madrigal et al. (2019) | 52 | Yes | Can't tell | Yes | Can't tell | Yes |
| Paterno et al. (2014) | 55 | Yes | Can't tell | No | Can't tell | Yes |
| Samari et al. (2020) | 57 | Yes | Can't tell | Yes | Can't tell | Yes |
| Stulberg et al. (2020) | 68 | Yes | Can't tell | Yes | Can't tell | Yes |
| **5. Mixed Methods** | | | | | | |
| **Author and Year** | **Citation Number** | **5.1 Is there an adequate rationale for using a mixed methods design to address the research question?** | **5.2 Are the different components of the study effectively integrated to answer the research question?** | **5.3 Are the outputs of the integration of qualitative and quantitative components adequately interpreted?** | **5.4 Are divergences and inconsistencies between quantitative and qualitative results adequately addressed?** | **5.5 Do the different components of the study adhere to the quality criteria of each tradition of the methods involved?** |
| Thorman et al. (2022) | 69 | Yes | Yes | Yes | Yes | Yes |
| Caskey et al. (2016) | 27 | Yes | Yes | Yes | Yes | Yes |
| Charron-Prochownik et al. (2014) | 29 | Yes | Yes | Yes | Yes | Can't tell |
| Dunlop et al. (2010) | 35 | Yes | Yes | Yes | Yes | Yes |
| Dunlop et al. (2013) | 36 | Yes | Yes | Yes | Yes | Yes |
